# Supplementary material for: Modular coherent photonic-aided payload receiver for communications satellites
Source: Nat Commun. 2019 Apr 30;10:1984. doi: 10.1038/s41467-019-10077-4 (PMC6491822; doi:10.1038/s41467-019-10077-4)
Supplement: Supplementary file 1 — Supplementary Information [file 41467_2019_10077_MOESM1_ESM.pdf]

Supplementary Information

Modular coherent photonic-aided payload receiver for communications satellites

Duarte, V. C. *et al.*

Supplementary Note 1: Stabilization provided by the Monitoring and Control Loop

The purpose of the monitoring and control loop (MCL) is to provide optimized beamforming steadily over time, which can be observed by monitoring the error vector magnitude (EVM) of the output signal over time. Such a test was conducted over 30 s with the same setup as depicted in Fig. 1a of the main text, and with deactivated and activated MCL. Results are shown in Fig. 1.

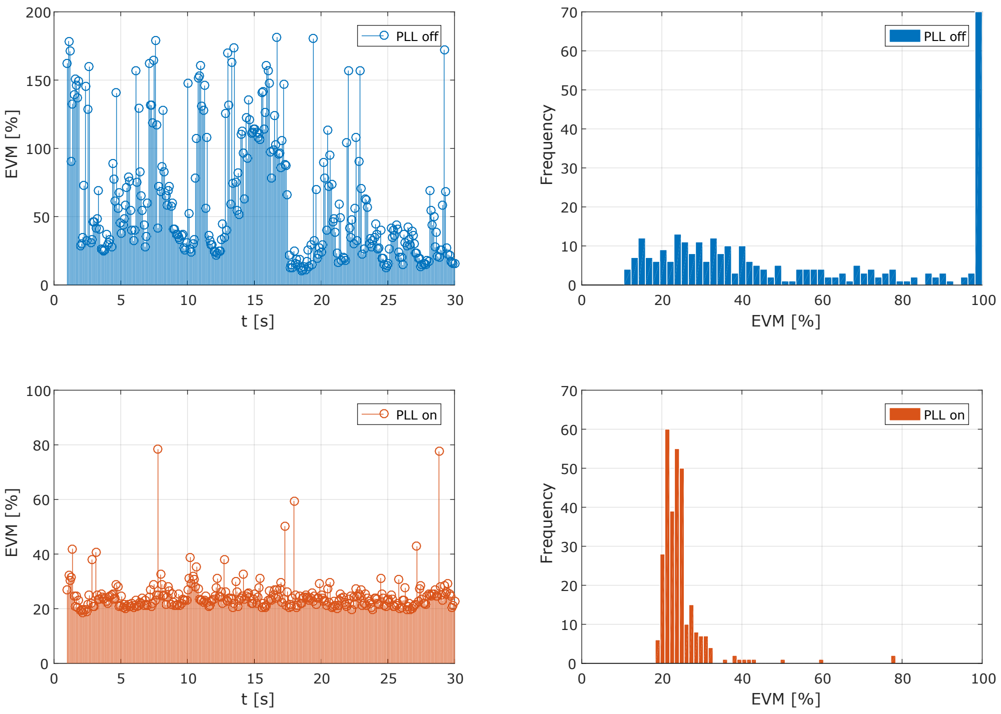

Supplementary Figure 1: **EVM variation over time when the MCL is deactivated (top) and activated (bottom).**

When the MCL is deactivated the phase of each signal wanders freely, which means that so does the EVM of the output signal. As a result, the system underperforms unacceptably most of the time. A minimum EVM of 10.5 % is achieved, marking the lowest possible EVM that can be reached. The reasons for being the lowest possible value are the following. First, dithering tones are deactivated, which means that weak but parasitic phase modulation does not impair the quadrature phase-shift keying (QPSK) signal. Second, the phase shifters β are biased at $(V_{\mathrm{max}} + V_{\mathrm{min}})/2 = -1.1\,\mathrm{V}$, which results in a constant voltage-dependent attenuation of 3 dB in all signals. Therefore, when the signals happen to be aligned in phase, best-performing beamforming takes place.

When the MCL is activated the EVM is stabilized to an average value of 22 %, which demonstrates correct phase-locked loop (PLL) operation, albeit with a performance penalty and with sporadic outliers. This also confirms that the period of the MCL of 100 ms is sufficient to track phase wandering. As explained in the previous paragraph, the performance penalty is caused both by the parasitic phase modulation imposed by the dithering tones, and also by a variable voltage-dependent attenuation imposed by the phase shifters β. Such an attenuation can reach 1.2 dB for a bias voltage of $V_{\mathrm{max}} = -0.7\,\mathrm{V}$. The fluctuating EVM around its average value is also due to voltage-dependent attenuation imposed by the phase shifters β. An outlier occurs whenever there is a reset of at least one phase shifter β to $(V_{\mathrm{min}} + V_{\mathrm{min}})/2 = -1.1\,\mathrm{V}$, corresponding to an abrupt phase variation of $\pm\pi$. As a result, beamforming gets disrupted until the MCL converges to phase equalization amongst signals. Resetting can be avoided using endless phase shifters, similarly to endless polarization controllers [1]. An endless phase shifter comprises at least two independent phase shifters, each with a dynamic range of 2π. Nonetheless, the disruption caused by the reset of a phase shifter decreases with N.

Supplementary Note 2: Impact caused by dithering tones

The previous section shows that dithering tones impose a weak but parasitic phase modulation. In this section, such a parasitic phase modulation is characterized. In order to do so, the real-time sampling scope (RTSS) was triggered with the generation of the dithering tones, configured with a sampling frequency of 6.25 $\mathrm{Gsa\,s^{-1}}$, and with a total time window of 8 ms. Dithering tones with different amplitudes were experimented. The dithering tones were measured to run for about 2 ms. Results are shown in Fig. 2.

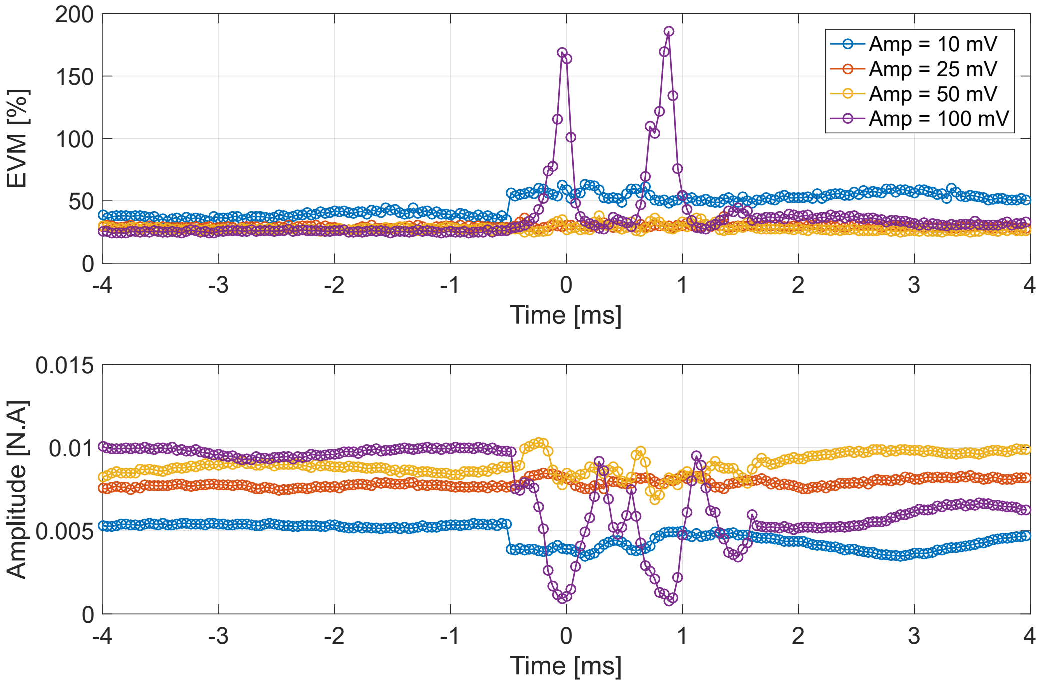

Supplementary Figure 2: **EVM and amplitude of the output signal over time.** Dithering tones are activated at around 0 s, with different amplitudes for each curve.

In terms of EVM, a slight disruption of both EVM and amplitude is observed even for the default amplitude of 50 mV. Such a disruption becomes practically negligible when the amplitude is reduced to 25 mV. The MCL fails to perform at the lowest amplitude of 10 mV, as the EVM increased instead of at least stabilizing. Setting the highest amplitude of 100 mV results in a severe disruption of the output signal.

As a conclusion, for the presented system the amplitude of the dithering tones should be between 25 and 50 mV.

Supplementary Note 3: Detailed description of the experimental setup

In this section, we provide the details of the devices and equipment used in the experimental setup. A tunable laser source (TLS) made by Yenista (OSICS model TLS-WDM) with a linewidth of 100 kHz and a wavelength of 1548.9 nm is used as the light source. The laser is connected to a polarization maintaning (PM) splitter with four outputs, two of which feed the two Mach-Zehnder modulator (MZM) arrays, and another feeding an IQ modulator that generates the frequency-shifted optical local oscillator (FSOLO). Each MZM array performs polarization multiplexing of the two output signals in order to simplify packaging, since this way only one output fibre is required.

The RF signal that is fed to the MZM arrays is generated by an arbitrary wave generator (AWG) (Keysight model M8195A), that has a bandwidth of 20 GHz and a maximum sampling rate of 64 Gsa s^{-1}. The amplitude of the RF signal is set to the maximum value of 1 V. Both differential outputs of the AWG channel generating the RF signal are used, each split into two copies, resulting in a total of four copies. The four RF amplifiers used for amplifying the four copies of the RF signal are broadband amplifiers (2×SHF model 810 and 2×SHF model 806E). While narrowband amplifiers for the used band would have been preferable, only broadband amplifiers were available. The signal generator that produces a tone of 26 GHz is a Rohde & Schwarz model SMR50. The programmed amplitude of the RF tone is of 0.5 V. The generated tone is split into two copies, one of which is phase-shifted by approximately 90° using a tunable phase shifter (Spectrum model LS-0140-KFKM). The resulting signals are amplified by broadband amplifiers (SHF model 807), and fed to an IQ modulator (u2t photonics, model SCMO2125). An optical carrier suppression relatively to a sideband of at least 15 dB is achieved by the MZM arrays. As for the FSOLO, the RF tone is at least 25 dB above other tones.

The two Erbium-doped fibre amplifiers (EDFAs) serving as pre-amplifiers are part of an Exelite Innovations model XLT-CFA-16, containing two EDFAs sharing the same pump. The signal generated by the IQ modulator is amplified by an EDFA from IPG, model EAD-1K-C3-W. The optical filters placed after the pair of EDFAs are identical commercial off-the-shelf (COTS) dense wavelength division multiplexing (DWDM) optical filters with a bandwidth of 100 GHz and a center wavelength of 1549.32 nm. The detuning between the optical filters and the laser wavelength of about 0.42 nm is beneficial, as it helps further suppressing the RF sideband that is not processed as well as the residual optical carrier. The Erbium-doped multicore fibre amplifier (EDMCFA) comprises seven cores organized in an hexagonal disposition, with core 7 as the center core. For the remaining six cores, core numbering is defined as clockwise, i.e., core k is adjacent to core $k+1$. All cores of

the EDMCFA share the same set of pumps, the power of which is split evenly by all cores. A bidirectional pumping scheme is employed. The noise figure of all cores of the EDMCFA was measured, and is between 5.5 dB and 6.5 dB. The variable optical attenuators (VOAs) used for power equalization rely on microelectromechanical systems (MEMS) technology, and are all made by Santec, model MOVA-HC. Fibre interfacing with the photonic integrated circuit (PIC) is done via grating couplers and a 16-channel V-Groove assembly with 127 µm spacing and a 16° angle chip surface made by OZ Optics. All polarization controllers are COTS manual three-paddle controllers. The transimpedance amplifier (TIA) is made by Analog Devices (model ADN2820), and has a bandwidth of 9 GHz and a gain of 5000 V A^{-1}. All horn antennas are made by Ocean Microwave, model OLB-28-10, and have a gain of 9.7 dBi at 28 GHz. Each antenna is 19 mm wide. This explains the considered distance between two adjacent antenna elements of 20.1 mm, corresponding to a margin of only 1.1 mm.

The MCL is implemented resorting to the following COTS devices. Each tone $f_{1,\mathrm{LO}}$ or $f_{2,\mathrm{LO}}$ is generated by a demonstration board from Linear Technology, model 1959A, based on the frequency synthesizer LTC6948. The RF IQ demodulator is a demonstration board made by Linear Technology, model DC1662A, based on the IQ demodulator LTC5585. The analog-to-digital converter (ADC) is made by Microchip Technology Inc., model MCP3903, and is placed in an evaluation board already containing the digital signal controller (DSC), which is from the model family dsPIC33F. The digital-to-analog converters (DACs) are made by Analog Devices (model AD5754). Each buffer comprises an operational amplifier (Texas Instruments OPA191) followed by fast power buffer (Linear Technology LT1010).

Data acquisition was handled by a RTSS from Tektronix model DPO72004 B, with a bandwidth of 20 GHz, a maximum sampling rate of 50 Gsa s^{-1} and an effective number of bits of 5.5 bit at 50 mV div^{-1}. All offline processing and communication between computer and AWG, RTSS and DSC is performed via Matlab, using universal serial bus (USB) communication. The EVM of the signal obtained in electrical back-to-back, i.e., AWG directly connected to the RTSS, was of 7.5 %.

The generation and processing of the pilot tones requires synchronism among involved devices and equipment. Such synchronism is obtained using the output reference frequency signal of the Rohde & Schwarz model SMR50, with a frequency of 10 MHz, which is shared to the AWG, frequency synthesizing boards and DSC.

The average power at different points of the system is detailed in Table 1. For the bottom four lines, paths other than the enabled one are attenuated by at least 30 dB. Electro-optic modulation is very lossy (> 30 dB) for two reasons. First, there were some problems during the packaging of the MZM arrays, resulting in insertion losses higher than expected, of 12 dB and 15 dB. Second, the RF frequency of 28 GHz is beyond the 3 dB cutoff frequency of some of the modulators (23, 23.8, 28.4 and 29.2 GHz), and especially of the AWG. The choice of the used four of the seven cores of the EDMCFA was based on minimizing crosstalk among cores, as the difference in output power among different cores is negligible. Nonetheless, crosstalk produced by the EDMCFA or even by polarization multiplexing and demultiplexing was observed to be negligible. The total insertion loss of a single path accounts for the efficiency of the input and output grating couplers ($\sim 2 \times 4.5$ dB), the loss of the phase shifter β (~ 2 dB), the loss of the phase shifters $\phi_{1,2}$ (~ 10 dB), the loss of the phase shifters $\gamma_{1,2}$ (~ 2 dB), and the inherent loss of the combining network of 6 dB. All of these losses amount to 29 dB. The remaining losses (~ 6 dB) are waveguide propagation losses (~ 1 dB cm^{-1}), and mainly the result of non-ideal alignment between fibre array and grating couplers.

Supplementary Table 1: **Average optical power at different points of the system.** Paths 1 and 4 correspond to the upper and lower signal path depicted in Fig. 1a of the main text.

**Point of the system**	**Power [dBm]**	**Comment**
Laser output	+9.99	Programmed value
Output of modulator array 1	−35.8	Input of EDFA 1
Output of modulator array 2	−28.7	Input of EDFA 2
Output of IQ modulator	−13.6	Input of EDFA 3
Output of EDFA 1	+8.8	Gain of 44.6 dB
Output of EDFA 2	+3.9	Gain of 32.6 dB
Output of EDFA 3	+6.0	Gain of 19.6 dB
Output of optical filter 1	−8.1	Filter located at the output of EDFA 1
Output of optical filter 2	−4.2	Filter located at the output of EDFA 2
Input of EDMCFA of path 1	−16.3	Core no. 1
Input of EDMCFA of path 2	−15.9	Core no. 5
Input of EDMCFA of path 3	−15.1	Core no. 6
Input of EDMCFA of path 4	−15.5	Core no. 2
Output of EDMCFA of path 1	+9.9	Gain of 26.2 dB
Output of EDMCFA of path 2	+9.9	Gain of 25.8 dB
Output of EDMCFA of path 3	+11.6	Gain of 26.7 dB
Output of EDMCFA of path 4	+11.2	Gain of 26.7 dB
Output of VOA of path 1	+9.4	VOA attenuation set to 0 dB
Output of VOA of path 2	+9.7	VOA attenuation set to 0 dB
Output of VOA of path 3	+11.3	VOA attenuation set to 0 dB
Output of VOA of path 4	+10.1	VOA attenuation set to 0 dB
Optical output of the PIC: path 1 enabled	−27.4	Total insertion loss of 36.8 dB
Optical output of the PIC: path 2 enabled	−23.7	Total insertion loss of 33.4 dB
Optical output of the PIC: path 3 enabled	−24.1	Total insertion loss of 35.4 dB
Optical output of the PIC: path 4 enabled	−25.1	Total insertion loss of 35.2 dB

Supplementary Note 4: Details of the photonic integrated circuit

The PIC was produced at the foundry of IHP in a multi-project wafer run, using the proprietary technology SG25_PIC. The layout and a photograph of the PIC is shown in Fig. 3.

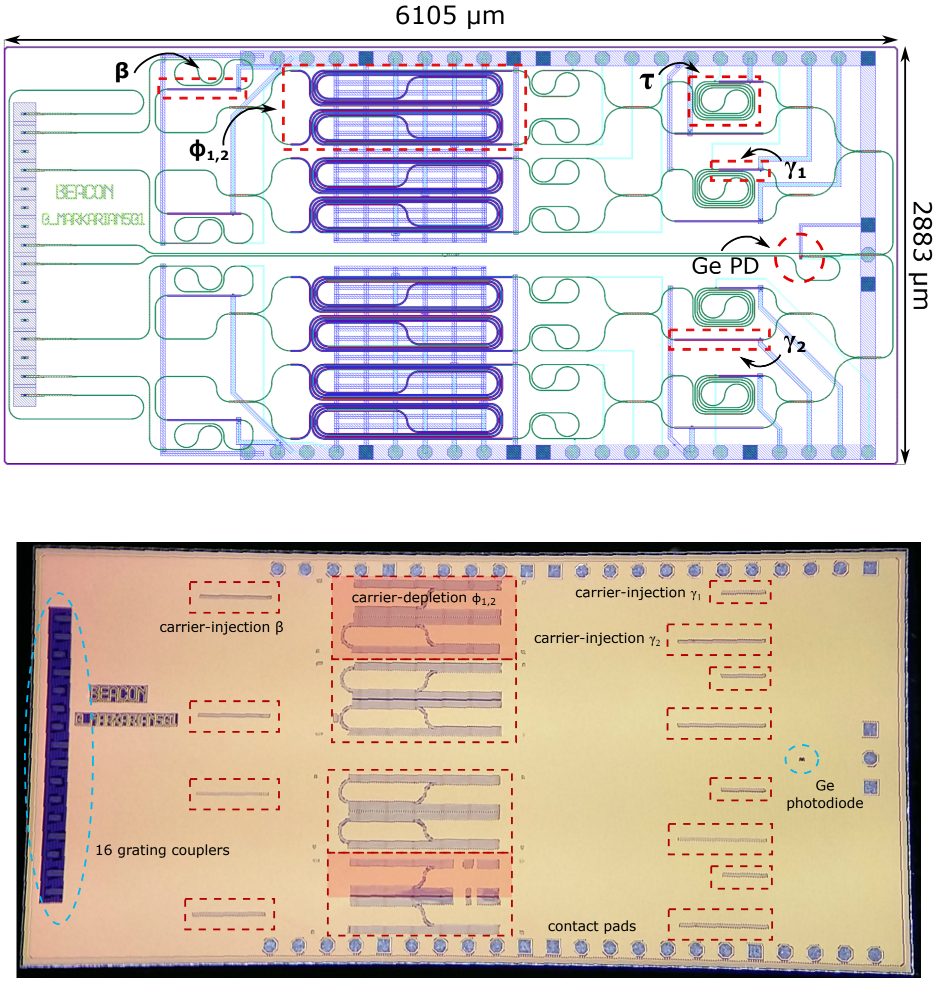

Supplementary Figure 3: **PIC layout and photograph, showing that some modules are at least partly visible at the surface.**

From left to right, each path starts with a carrier-injection phase shifter 500 µm long (β), followed by a tunable optical delay line (TODL), a combiner network and a Germanium photodiode. The TODL is a Mach-Zehnder delay interferometer (MZDI) with variable coupling ratio and with a tuning range of $\tau = 50\,\text{ps}$. The operation principle of the TODL is detailed in [2], and an experimental demonstration including the MCL is reported in [3]. The extensive description already provided in both articles combined with the fact that the short tuning range of the TODLs makes these of little use to the present demonstration are the two main reasons why the explanations behind the TODLs are made concise in the present article. The composition of each TODL is as follows. A pair of coiled carrier-depletion phase shifters with 1 cm ($\phi_{1,2}$) is used to vary the coupling ratio of the MZDI, thus allowing to vary the delay of the TODL from 0 to τ. A pair of carrier-injection phase shifters, one with 300 µm (γ_1) and the other with 600 µm (γ_2), is required to center the frequency response of the MZDI with the center frequency of the delayed signal [2]. While the pair of carrier-depletion phase shifters $\phi_{1,2}$ are designed to facilitate push-pull operation [3], such is not the case of the pair of carrier-injection phase shifters $\gamma_{1,2}$. The phase shifter γ_1 is not used, and serves to minimize the loss difference between its path and the path of the phase shifter γ_2.

There are also thermo-optic phase shifters in series with the phase shifters β, $\phi_{1,2}$ and γ_1, designed as fallback solutions. The latter consists of a resistive track designed on top of the coiled delay line (τ). None of these are used. The reason why carrier-depletion phase shifters were considered for $\phi_{1,2}$ instead of more compact carrier-injection ones was to verify whether there would be any issue in integrating all three common kinds of silicon photonics phase shifters in the same PIC. Such turned out not to be a problem.

During initial characterization tests, three carrier-depletion phase shifters did not respond to an applied voltage. These are highlighted in the figure. The cause behind such failure was found out to be a design error in the metal layers, cutting off electrical access to the phase shifters. The consequence of such error is that the TODL of the upper path cannot be

tuned. Nevertheless, such does not pose any constraint, as the upper path may serve as the time reference to which the time delays of the remaining three paths are tuned. While the lowest path only has one operating carrier-depletion phase shifter, the corresponding TODL could be tuned in its entire range. The carrier-depletion phase shifters were designed with a long length for precaution in order to allow correct TODL operation even if one of the phase shifters failed to operate, thus having a dynamic phase range of 2π.

The behavior of the three kinds of phase shifters is identical to that described in Fig. 4 of [3], even though the length of the phase shifters used in the present work is different from those reported in the cited article. The differences are the following. The carrier-injection phase shifters β reach a maximum phase-shift of 2π when biased at $-1.5\,\mathrm{V}$. The voltage-dependent attenuation smoothly increases as the bias voltage decreases, reaching about 6 dB for a minimum bias voltage of $-1.5\,\mathrm{V}$. The phase dynamic range of the coiled carrier-depletion phase shifters is of 2π for a bias voltage varied between $-0.7\,\mathrm{V}$ and $6\,\mathrm{V}$. The voltage-dependent attenuation smoothly increases as the bias voltage decreases, reaching about 2.3 dB for a minimum bias voltage of $-0.7\,\mathrm{V}$.

Characterization tests of the Germanium photodiode revealed a 3 dB cutoff frequency of 42 GHz, a dark current below 50 nA and a responsivity of $0.85\,\mathrm{A\,W^{-1}}$.

Supplementary Note 5: Offline digital signal processing

The RTSS is connected to one of the outputs of the TIA for sampling the RF signal that is output by the system. By default, the RTSS outputs a frame that is 640 symbols long, i.e., as long as the frame produced by the AWG. The sampled QPSK signal, at a down-converted carrier frequency of 2 GHz, is processed in Matlab according to the following steps. First, down-conversion to baseband is performed, followed by rectangular filtering to remove pilot tones and excess noise. Then, down-sampling to 1 sample per symbol is performed. The chosen sampling point is the one that produces the sampled signal with minimum variance. The amplitude is normalized and the constellation is de-rotated by a constant angle for all symbols. This means that no carrier phase recovery algorithm was used to mitigate any kind of phase noise or parasitic phase modulation.

Concerning figures of merit, the EVM is measured as described in [4], with the reference symbols obtained from applying hard-decision to the input symbols. The symbol error rate (SER) of a given sequence is estimated after time-aligning such a sequence with the original sequence, experimenting all possible rotations of $\pi/2$, and selecting the one which minimizes the SER. The presented values of amplitude, EVM and SER are the average of 100 frames. The representative constellations are the overlap of the constellations of the best frames for every possible combination, i.e., paths 1, 2, 3 and 4 for one enabled path; paths $1+2$, $2+3$, $3+4$, $1+3$, $2+4$ and $1+4$ for two enabled paths; and all four enabled paths.

Supplementary Note 6: Assessment of custom-made modules in a space environment

Upscaling and implementing the proposed photonic payload receiver in a communications satellite must be done step by step, by increasing the technology readiness level from the current level 3 up to level 7. Such implies not only upscaling the proposed system, but also space-qualifying components, sub-systems and, finally, the complete system. Space qualification is a meticulous process that aims to guarantee that the qualified devices and systems are able to operate reliably in the harsh conditions of space for the entire lifetime of the satellite. In this section, we summarize the results obtained from trials conducted in collaboration with ALTER test house in CNA (Centro Nacional de Aceleradores) Gamma Facility in Seville, Spain, with the objective of assessing the behavior of GaAs modulators and custom-made Erbium-doped fibres (EDFs) to space conditions.

The environmental credentials of GaAs/AlGaAs III-V semiconductor material system on which the modulator arrays build are well known. It has many desirable properties for RF devices which must survive and operate in harsh environments, and has remained the material of choice for mm-wave electronics and monolithic microwave integrated circuits. It is thus a natural choice for space-borne systems. Nonetheless, four IQ modulators fabricated and packaged with the same methods as the modulator arrays used in the experiments were put to test. The conducted trials were the following: endurance to a temperature of 70 °C for 2000 h; gamma irradiation at a rate between 3.6 and $36\,\mathrm{krad\,h^{-1}}$ up to a total ionizing dose (TID) of 100 krad; random vibration during 3 min per axis; shock of 500 g in 1 ms; proton flux at 60 meV up to $10^{12}\,\mathrm{cm^{-2}}$; and 50 thermal cycles between $-40\,°\mathrm{C}$ and $85\,°\mathrm{C}$ under vacuum. The performance metrics were insertion loss, extinction ratio and half-wave voltage, V_π. Alter did not observe consistent variations in the measured metrics, concluding that degradation in performance was not observed.

Concerning the EDMCFA, an EDF identical to the one of the used amplifier was subjected to gamma irradiation up to a TID of 100 krad. A radiation-induced gain drop of only 0.7 dB was measured with a pre-irradiation gain set to 26.1 dB, showing negligible performance degradation [5]. All the remaining components of the EDMCFA, i.e., laser pump diodes and passive components, did not require testing as it were already space-qualified or in the process of being so.

No tests were done to PICs, as IHP's technology SGB25RH, similar to the one used for fabricating the PIC, was under evaluation by the European Space Agency targeting space qualification. Such evaluation was successfully concluded [6]. No tests were done to any part of the MCL, as it was implemented with COTS modules not suitable for a space environment.

In summary, preliminary trials for assessing custom-made modules in a space environment did not show significant degradation in performance. Ensuing space qualification looks promising.

Supplementary References

[1] Noe, R., Heidrich, H. & Hoffmann D. Endless polarization control systems for coherent optics. *Journal of Lightwave Technology* **6**, 1199-1208 (1988).

[2] Duarte, V. C., Drummond, M. V. & Nogueira, R. N. Photonic True-Time-Delay Beamformer for a Phased Array Antenna Receiver based on Self-Heterodyne Detection. *Journal of Lightwave Technology* **34**, 5566-5575 (2016).

[3] Duarte, V. C., *et al.* Reconfigurable monitoring and control system for tunable optical delay lines. *Optics Letters* **43**, 2543-2546 (2018).

[4] Shafik, R. A., Rahman, M. S. & Islam, H. M. R. On the extended relationships among EVM, BER and SNR as performance metrics. *2006 International Conference on Emerging Technologies*, 27-31 (2006).

[5] Filipowicz, M. *et al.* Radiation-hardened optical amplifier based on multicore fibre for telecommunication satellites. *SPIE Technologies for Optical Countermeasures XIV*, **104350C** (2017).

[6] IHP technology ready for space flights [Press release]. Available at: `https://www.ihp-ffo.de/uploads/media/PM_2018-08-20_EPPL-Liste_eng.pdf` [Accessed 10 Dec. 2018].
